# Influence of alkaline modification on selected properties of banana fiber paperbricks

**DOI:** 10.1038/s41598-021-85106-8

**Published:** 2021-03-11

**Authors:** Abayomi A. Akinwande, Adeolu A. Adediran, Oluwatosin A. Balogun, Oluwaseyi S. Olusoju, Olanrewaju S. Adesina

**Affiliations:** 1 Department of Metallurgical and Materials Engineering, Federal University of Technology, Akure, Ondo State Nigeria; 2 Department of Mechanical Engineering, Landmark University, PMB, 1001, Omu-Aran, Kwara State Nigeria; 3 Faculty of Material Science and Chemical Engineering, Ningbo University, Ningbo, China

**Keywords:** Structural materials, Materials science, Engineering, Civil engineering

## Abstract

In a bid to develop paper bricks as alternative masonry units, unmodified banana fibers (UMBF) and alkaline (1 Molar aqueous sodium hydroxide) modified banana fibers (AMBF), fine sand, and ordinary Portland cement were blended with waste paper pulp. The fibers were introduced in varying proportions of 0, 0.5, 1.0 1.5, 2.0, and 2.5 wt% (by weight of the pulp) and curing was done for 28 and 56 days. Properties such as water and moisture absorption, compressive, flexural, and splitting tensile strengths, thermal conductivity, and specific heat capacity were appraised. The outcome of the examinations carried out revealed that water absorption rose with fiber loading while AMBF reinforced samples absorbed lesser water volume than UMBF reinforced samples; a feat occasioned by alkaline treatment of banana fiber. Moisture absorption increased with paper bricks doped with UMBF, while in the case of AMBF-paper bricks, property value was noted to depreciate with increment in AMBF proportion. Fiber loading resulted in improvement of compressive, flexural, and splitting tensile strengths and it was noted that AMBF reinforced samples performed better. The result of the thermal test showed that incorporation of UMBF led to depreciation in thermal conductivity while AMBF infusion in the bricks initiated increment in value. Opposite behaviour was observed for specific heat capacity as UMBF enhanced heat capacity while AMBF led to depreciation. Experimental trend analysis carried out indicates that curing length and alkaline modification of fiber were effective in maximizing the properties of paperbricks for masonry construction.

## Introduction

Buildings are structures with roofs and walls constructed in different shapes, sizes, and dimensions which purposes are to meet societal needs in terms of shelter, security, office spaces, and other relevant purposes^1,2^. Different materials are used for building construction ranging from bamboo, woods, plastics, clay bricks: both fired and unfired, and concrete bricks^3–7^. The use of each aforementioned material has its own merits and demerits. Some of the advantages are high strength, water resistance, and stability. Demerits may include high cost, poor thermal insulation, and high density.

Paperbrick or papercrete brick is a newly developed building material that has good thermal and sound insulation as an advantage^8,9^. These properties are needed for buildings nowadays based on noise pollution and high temperatures experienced owing to climate change. The concept of developing these paperbricks is hinged on recycling of waste paper, since papers are used in large quantities daily, all over the world^10,11^. Other gains in paperbricks are lightweight, low energy consumption during the production process, low cost, and ease of transportation as compared to conventional bricks. Paperbricks are produced by the mixture of cement, paper pulp, sand, and other forms of additives in varying proportions towards satisfying certain specifications^12^. Paperbricks have little patronage for building purposes owing to the low strength acclaimed to them and high water absorption tendency since paper is susceptible to water assimilation. One important input in brick production is ordinary Portland cement. Cement has been proved to be a good binder^13^ with a tendency for enhancing bond strength within the matrix, a concept utilized in concrete and other cement composites. The curing process is an important process employed in concrete development towards strength improvement^14^. Investigations carried out by^15,16^ revealed an enhancement of strength from 7 to 28 days of curing. Correspondingly, reports were recorded in^17,18^ and the endpoint is the heightening of strength with curing age. With the right curing environment, strength is enhanced over curing days based on continuous hydration process amounting to formation of calcium silicate hydrate; responsible for long-term strength of cement matrix^19^.

Sandcrete brick majorly have sand as the matrix with cement as the binder and strengthener. In Nigeria and most African countries, hollow blocks used in building are mainly produced by the mixture of sand and cement in adequate proportions^20,21^. These bricks are characterized by relatively good strength but are heavy and of high cost. If such bricks produced from the admix of cement and sand are employed in building applications, then a trial on the properties of cement, sand, and paper (matrix) mixture is worth investigating towards developing lightweight and cheaper paperbricks for masonry, which can be used for low-cost bungalows or in the building of stable make-shift homes (instead of using tents) for internally displaced persons (IDP) in dry regions like a desert; where rainfall is scarce. Note that concrete blocks, been ceramic materials exhibit brittle failure under quasi-static loading and this kind of failure is catastrophic^22^ for building structures. Furthermore, concrete bricks exhibit lower impact strength, which is identified in the scattering of these bricks when they fall from a “low” height^23^. Paperbricks on the other hand, due to cellulose fiber inherent, have the tendency of showcasing improved impact resistance and exhibit a type of plastic deformation in the fracture region.

Fiber of natural and synthetic type have been employed by different researchers in enhancing the toughness of bricks. Authors^24^ employed banana fiber in strengthening concrete, from the results obtained, the compressive strength was enhanced from 0 wt to 15 wt% banana fiber. Likewise ^25^, applied banana fiber in reinforcing conventional concrete and observations made was that 28th-day compressive strength appreciates up to 0.4 wt. addition. 28th-day tensile strength was noticed to improve with increasing fiber loading. Banana stem fiber was also observed to amplify the compressive strength of concrete at 3, 7, and 28 curing days in the investigation carried out in^26^. In the same way, tensile and flexural strengths followed the same pattern for 3, 7, and 28 days. The study further revealed improvement of impact and bond strengths at higher fiber loading from 0 to 2.5 wt%. It was also observed that strength properties appreciated with curing length (days), identifying the place of curing in the strength amplification of cement composites.

Additionally, inquiry into the strength properties of concrete in^27^ unfolded uptrend in compressive, splitting tensile, and flexural strength as curing time was lengthened. On the premises of these highlighted outcomes, banana stem fiber was adopted in this study to further enhance the properties of paperbricks.

**Figure 1:**
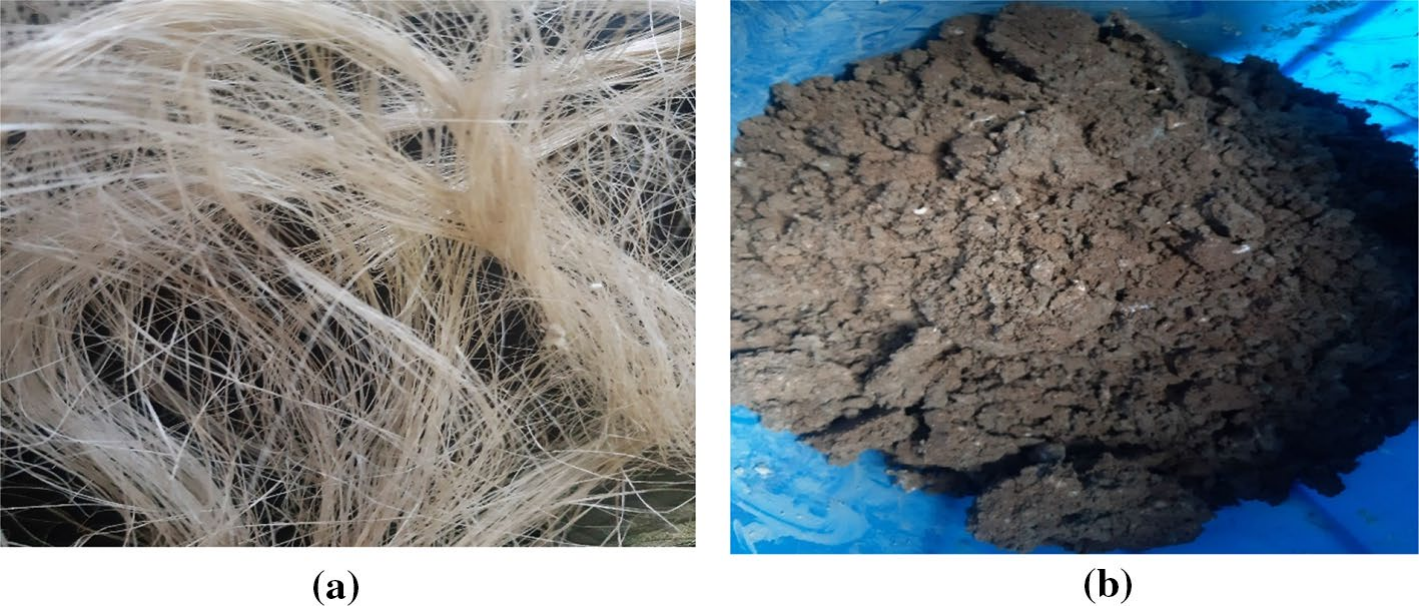
Image of materials used in the study (**a**) banana stem fiber (**b**) cement–sand–paper slurry.

Alkaline modification of fiber has been implemented in accentuating the strength performance of banana fiber as additives in bricks. Studies^28,29^ showed that alkaline modified banana fibers performed better than the untreated counterpart. Whence, this study aimed at reinforcing paperbricks with unmodified and alkaline modified banana stem fiber (Fig. 1) towards developing low-cost masonry bricks. Properties of composite units produced were assessed with the view to investigate the influence of alkaline modification of banana fiber. Furthermore, experimental trend and performance analysis was carried out by appraising the efficiencies of experimental inputs which are fiber addition, alkaline modification, and curing duration as developed and utilized in^30,31^.

### Materials preparation and development

Waste paper cardboard was initially shredded and soaked in a container of water for 7 days after which the water was dripped off. This was succeeded by mechanical blending. Paper slurry produced was transferred into a sack and subjected to a pressure of 10 MPa to allow total dripping of water from the slurry. The pulp was sundried for 5 days and packed for specimen preparation. Banana fibers were washed and a portion was treated with 1 Molar aqueous sodium hydroxide for 6 h, washed with distilled water, maintained at 40 °C, and dried in open air for 24 h. The other portion was left untreated.

Other materials used for fiber-paperbricks development are cement and sand sieved to − 75 µm. Sand was collected from a riverbank and treated by adding water and stirred, after which it was allowed to settle for 2 days. Water at the top layer was poured off and freshwater was introduced, stirred, and the mixture passed through a sieve of mesh 1.18 mm. The sand collected was dried in an oven for 24 h after which, dried sand was milled. Milled sand was then sieved by applying an electric laboratory sieve shaker whose upper aperture was 4.75 mm ASTM D6913M-17^32^. Fine sand collected on the pan (− 75 µm) was used as a particulate additive in the matrix.

Paper pulp, fine sand, and cement were blended and mixed (water: cement ratio was 0.42) in a locally fabricated tow mixer to form paper cement slurry. The first group of samples were produced by blending unmodified banana fiber (UMBF) into the slurry and the second group was produced the same way with alkaline modified banana fiber (AMBF) in the proportions illustrated in Table 1. Wastepaper slurry product was transferred into cuboid moulds which dimensions are 400 × 100 × 100 (mm^3^) and 190 × 90 × 90 (mm^3^), cylindrical moulds of diameter 100 mm and height 200 mm, and cube moulds of length 100 mm. The mixture was compacted in each mould applying a pressure of 5 MPa. Fresh samples produced were immediately transferred into a polymer container placed in a water basin and covered. Samples were cured in the container for 28 and 56 days after which they were examined for different properties.

**Table 1:** Composition mix.

Components (by weight proportion of waste paper pulp)			
Banana fiber (wt%)	Cement (wt%)	Fine sand (wt%)	Paper waste pulp (wt%)
0	25	25	50.0
0.5	25	25	49.5
1.0	25	25	49.0
1.5	25	25	48.5
2.0	25	25	48.0
2.5	25	25	47.5

### Test methodology

**Figure 2:**
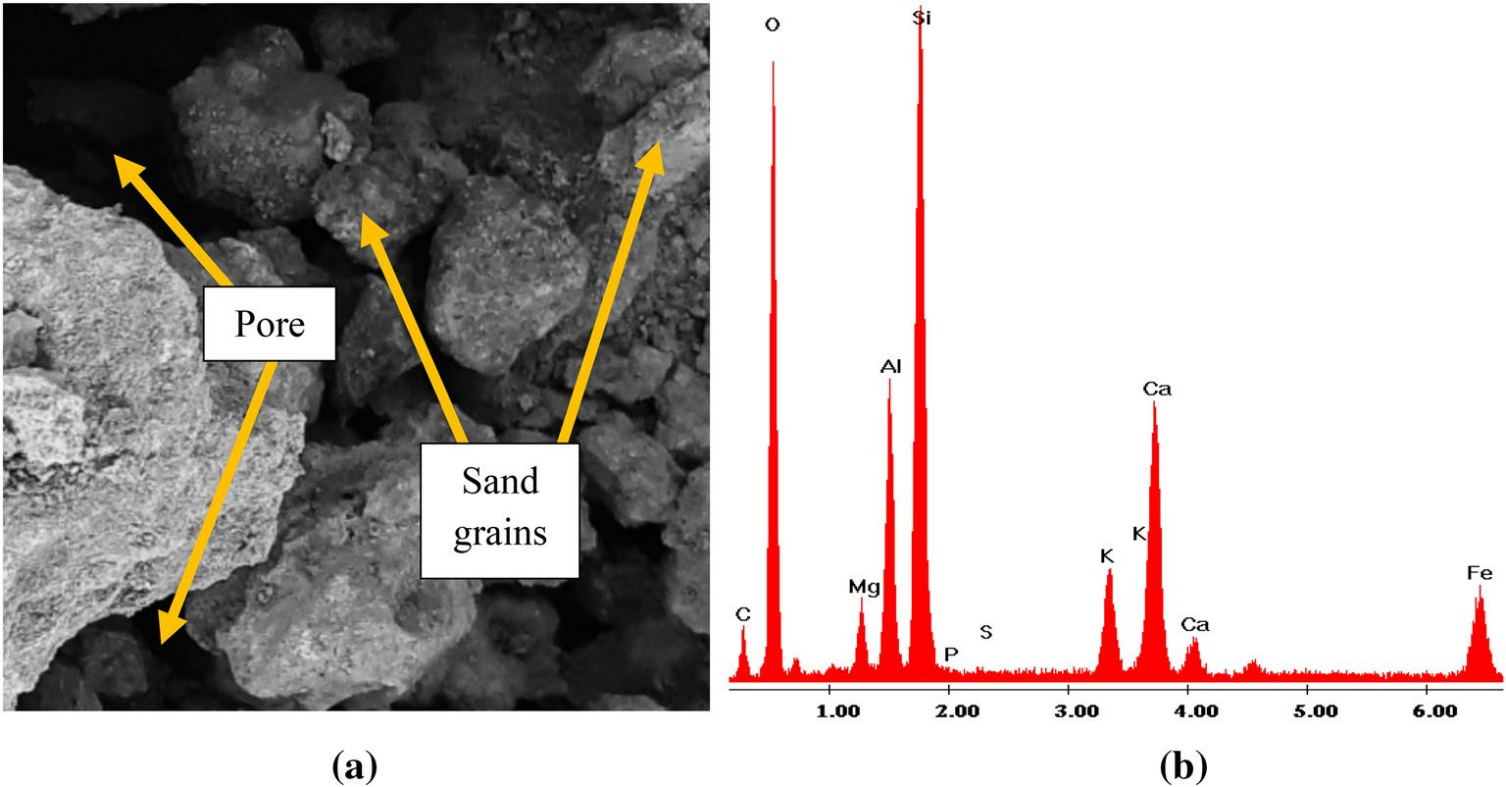
SEM micrograph and EDX of sand employed in the study.

**Figure 3:**
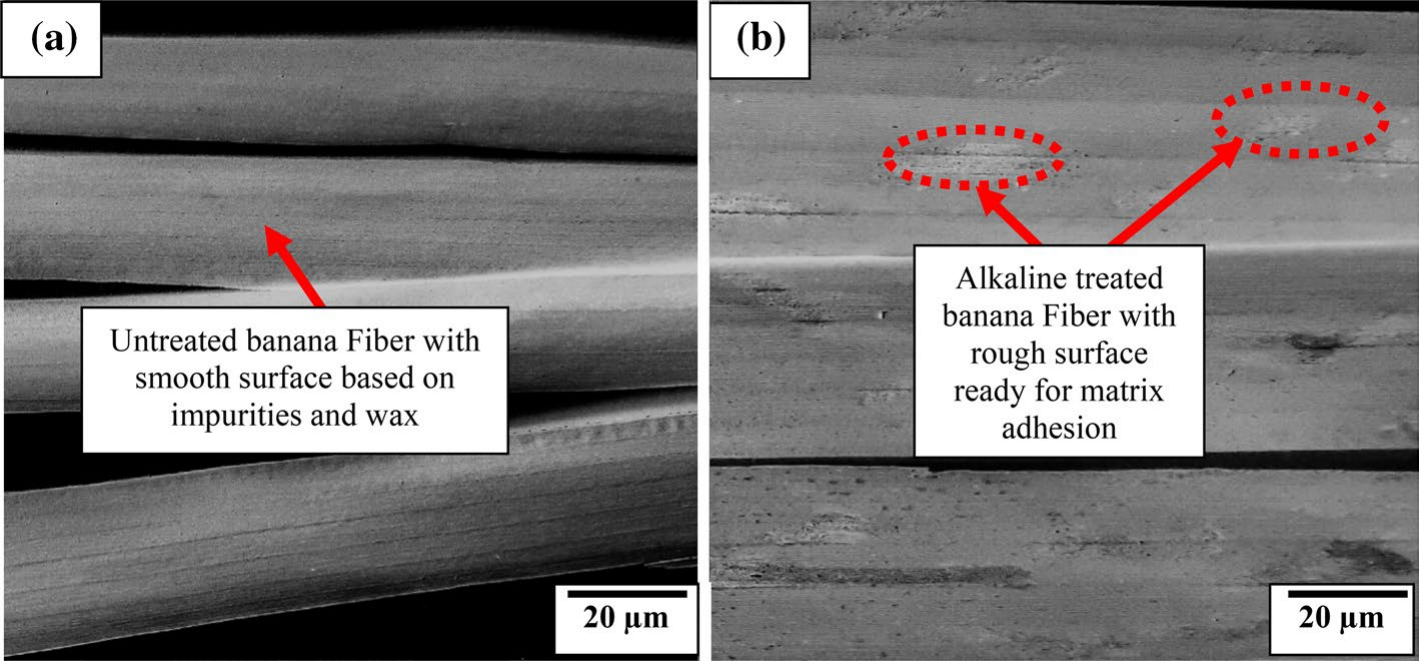
SEM Image of (**a**) untreated and (**b**) 1 M NaOH treated banana fiber.

A preliminary test was carried out on the materials used which include the chemical composition of sand, paper pulp, and banana fiber. Specific gravity, relative density, moisture content, and fineness modulus of the fine sand were tested in line with IS 2720 Part 3, BS 1377-2, IS 383–1970^33–35^, respectively. Morphology of the fine sand, unmodified banana fiber, and alkaline modified were also examined (Figs. 2 and 3). In carrying out the tests on the samples, each was dried in an autoclave at 40 °C for 12 h and the average of values obtained from testing three samples representing each mix proportion was recorded. Water absorption was investigated on samples of dimension 190 × 90 × 90 (mm^3^) in conformity with BS 1881-122, ASTM C 1585-20^36,37^. Initial weight in air (W_1_) was measured after which samples were soaked in water at 27 °C room temperature for 24 h. Weights of soaked samples were measured when suspended in air (W_2_). Moisture absorption was examined on samples of dimension 190 × 90 × 90 (mm^3^). Samples were initially dried in autoclave at 40 °C for 2 h and dried weight in air was taken as M_1_. Samples were exposed in the open air for 24 h, after which the masses in the air after 24 h moisture exposure was measured as (M_2_). Moisture absorption was therefore weighed using Eqs. (1) and (2).

1 $${\text{Water}}\;{\text{absorption}} = \frac{{ \left( {W2 - W1} \right) \times 100 }}{W1}$$

2 $${\text{Moisture}}\;{\text{absorption}} = \frac{{ \left( {M2 - M1} \right) \times 100 }}{ M1}$$

Compressive strength refers to the capacity of a material to resist compressive load and was investigated as stated in ASTM C39/C39M^38^ and the value was obtained employing Eq. (3). Samples (100 mm cubes) were subjected to compressive test using a universal testing machine at a loading rate of 30 kN/min. Three cube samples were examined for each mix proportion, with the samples placed centrally in the machine and the faces subjected to loading.

3 $${\text{Compressive}}\;{\text{strength}} = \frac{Maximum \;load \left( P \right)}{{cross\;sectional\;area \left( A \right) }}$$

Splitting tensile strength was examined on cylindrical samples of diameter 100 mm and height 200 mm according to procedures stipulated in ASTM C 496-17^39^ and the load was applied at a loading rate of 30 kN/min on the sides. The value was realized using Eq. (4).

4 $${\text{Splitting}}\;{\text{tensile}}\;{\text{F}}_{{\text{t}}} = \frac{2P}{{\pi Dl}}$$

where P is the load applied, D is the diameter of the cylindrical sample, and L is the length of the sample.

Flexural strength refers to resistance to bending stress exhibited by a material and was appraised via center point loading as indicated in ASTM 293/C293-M^40^. The test was carried out on samples of dimension 400 × 100 × 100 (mm^3^), with a span length of 300 mm. Parameters such as the maximum fracture Load (L) exerted at the center point of the sample, length (p) of the support plan, width (b) and the thickness (d) of the sample were recorded. Flexural strength was obtained using the expression in Eq. (5).

5 $${\text{Flexural}}\;{\text{strength}} = 3{\text{Lp/2bd}}^{2}$$

Thermal tests conducted are thermal conductivity and specific heat capacity. Thermal conductivity was examined employing the guarded hot plate method ASTM E 1225–13^41^ while evaluation of specific heat capacity was done as per ASTM E 1269–11^42^ via differential scanning calorimetry.

From the results obtained for all properties, property trend analysis and performance evaluation was carried out as developed in^30^ to determine the fiber addition efficiency, alkaline modification, and curing length (duration) efficiency, which are the three experimental variables (in this study) that determine the values of properties obtained for each mix proportion.

## Results and discussion

### Results of preliminary test carried out on input materials

Results of the preliminary test carried out on the materials used present the outcome of the SEM image of the natural sand employed showing pores and grains (Fig. 2a). EDX results on sand (Fig. 2b) indicate silicon and oxygen in high proportion which is also reflected in the chemical composition of the sand depicting silica content of 71.5% (by mass) (Table 2). Properties of the milled sand are highlighted in Table 3, where it is recorded that the sand has a fineness modulus of 1.65 indicating a good grading hence classified as fine sand following^35^.

**Table 2:** Chemical composition of milled sand.

Composition	SiO_2_	Al_2_O_3_	CaO	Na_2_O	MgO	Fe_2_O_3_	TiO_2_	K_2_O	MnO	Others	LOI
Amount (%)	71.5	15.6	0.41	1.34	0.21	2.11	0.89	0.15	1.94	4.64	1.21

**Table 3:** Physical properties of the milled sand.

Properties	Specific gravity	Relative density	Moisture content	Fine modulus
Sand	2.67	1.73	4.2	1.65

**Figure 4:**
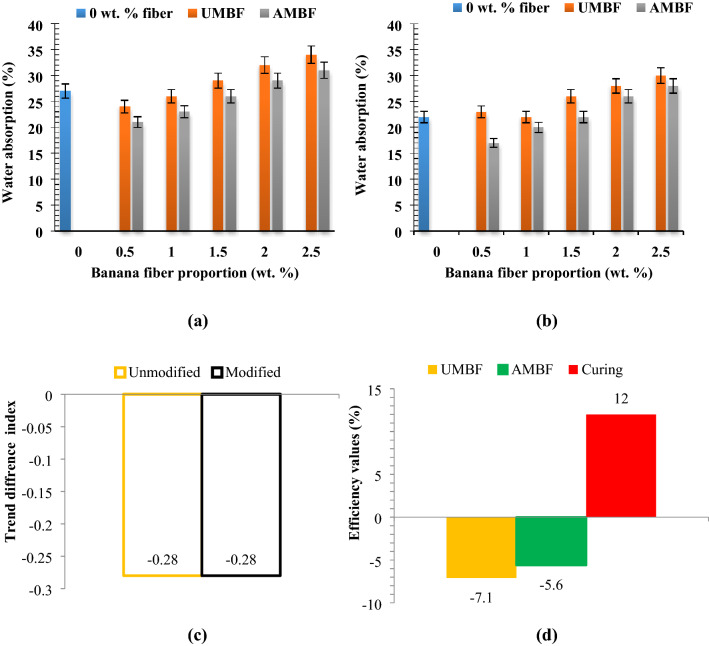
Relation between fiber proportion of unmodified and modified banana fiber and water absorption (**a**) 28 days and (**b**) 56 days with (**c**) experimental trend analysis and (**d**) property evaluation of experimental variables.

Figure 4a highlights the features present in untreated banana fiber. It is revealed that the fiber has a smooth surface due to wax and impurities attached to the surface. These impurities amount to poor adhesion between fiber and matrix^43^. Moreover, lignin and hemicellulose are reported to be responsible for the hydrophilic nature of the untreated fibers. Alkali treatment reduced the amount of lignin, hemicellulose content (Table 4) and resulted in dewaxing of the surface causing exposure of the rough surface of the fiber (Fig. 3b). The cellulose content of the fiber was modified resulting in depreciation in the hydrophilic tendency of banana fiber as shown in Table 4. Waste newspaper pulp as illustrated in Table 4 depicts the cellulose, hemicellulose, and lignin content present in the pulp.

**Table 4:** Chemical compounds present in waste paper, untreated and treated banana fiber.

	Paper pulp	Unmodified banana fiber (UBF)	Modified banana fiber (MBF)
Cellulose (%)	62	47.8	53.8
Hemicellulose (%)	21	23.5	19.7
Lignin (%)	10	25.6	23.8
Ash (%)	1.9	–	–
Others (%)	5.1	3.1	2.7

In the discussion of the results, untreated fiber-reinforced samples cured for 28 and 56 days are designated UMBF-28 and UMBF-56 accordingly, while those of treated samples are designated AMBF-28 and AMBF-56 for curing ages of 28 and 56 respectively.

### Analysis of the properties of samples

#### Water absorption

Water absorption in paperbricks can be linked-to the total porosity of the paper pulp coupled with the surface pores of fiber reinforcement. Water absorption (Fig. 4a,b) rate was revealed to rise with increased fiber loading for untreated and treated under both curing ages of 28 and 56 days. Nonetheless, it was noted that the water absorption rate in the AMBF samples is lower than in the UMBF samples, insinuating that water absorption is lesser with alkaline modified fibers than the untreated ones. The feat occurs on the dint of alkaline modification of banana fibers which led to cellulose restructuring and surface pore refinement^44,45^. Note that natural fibers are hydrophilic^46,47^ and as such have a tendency of absorbing water.

A clear observation made is that the water absorption rate decreased with higher curing age; from 28 to 56 days. This distinct observation is traceable to the hydration process which occurred up to 56 days. Hydration reaction results in the formation of hydrates (C-S–H, C-H) which begin with gel formation^48,49^. These hydrates spread into and over inherent pores, and on hardening presents a water-resisting bond within the matrix^50^. Implication of this is traceable to reduced porosity and water absorption rate. The utilization of paper as additives in cementitious composites intermixed with paper has shown that water absorption trended upward with increasing paper addition^51–53^. In a previous study of^54^, curing length was detected to minimize water absorption, while fiber addition promoted water assimilation as revealed in the present investigation. Water absorption indicated in this study seems slightly higher than the values reported in most research work carried out on concrete bricks^54^ which is due to the paper pulp matrix. Sand matrix in the case of concrete bricks has lower water absorption than paper pulp matrix as in the case of paperbricks. This explains why water absorption in concrete bricks is lesser than paperbricks. Howbeit samples infused with 1 and 2 wt% fibers which were cured for 56 days still fell within the standard requirements as enshrined in^55^. Natural fibers have been reported to be prone to water retention. Kraft paper fibers employed in^56^ revealed a consistent rise in water assimilation in the masonry brick developed. ^57^ examined coconut, oil palm, and bagasse fibers as potential building materials. The result of the water absorption test revealed that these fibers aroused a proportional increase in water absorption of the brick developed. Water uptake of untreated and alkaline treated abaca fiber was evaluated by^58^. Abaca fiber was dewaxed in ethanol, benzene and further treated in NaOH. Results of water absorption showed that both untreated and treated fiber absorbed water, nonetheless, the untreated absorb a higher volume of water when compared with the treated which is in tandem with the outcome argued in this study. The authors attributed the feat to the removal of lignin and hemicellulose components of the fiber via treatment, modifying the fiber. Conclusively natural fibers have hydrophilic tendencies but via treatment, the fibers are modified reducing the water retention capacity of the fibers. Further research can be directed towards the reduction of the water absorption either via coating of the surface with water-impermeable materials or review of the cement/sand content.

- Experimental trend analysis and performance evaluation on water absorption

Referring to Fig. 4c,d, the trend direction followed the same pattern in that, the values documented for the index are + 0.85 (UMBF-28) and + 0.78 (AMBF-28) affirming uptrend in water absorption for both kinds of fibers for samples cured for 28 days. As per UMBF-56 and AMBF-56, the values are + 0.57 and + 0.5, respectively, also affirming the uptrend under 56 days curing. When moving from 28 to 56 as per curing age the trend difference is − 0.28[59° acw] and − 0.28 [65° cw] for UMBF and AMBF respectively, where cw stand for clockwise and acw stands for anticlockwise. The analysis implies that on moving from 28 to 56 curing ages, there was depreciation in water absorption. Unmodified fiber addition increased water absorption slightly by 7.1% [ −] on the average while alkaline modification had a significant effect on water absorption with alkaline modified banana fiber having an efficiency of 5.6% [ −]. This shows that UMBF contributed more to water absorption than AMBF indicating alkaline modification of 1.5% [ +]. Curing efficiency of 12% [ +] shows that curing was 12% effective in reducing water absorption rate.

#### Moisture absorption (%)

**Figure 5:**
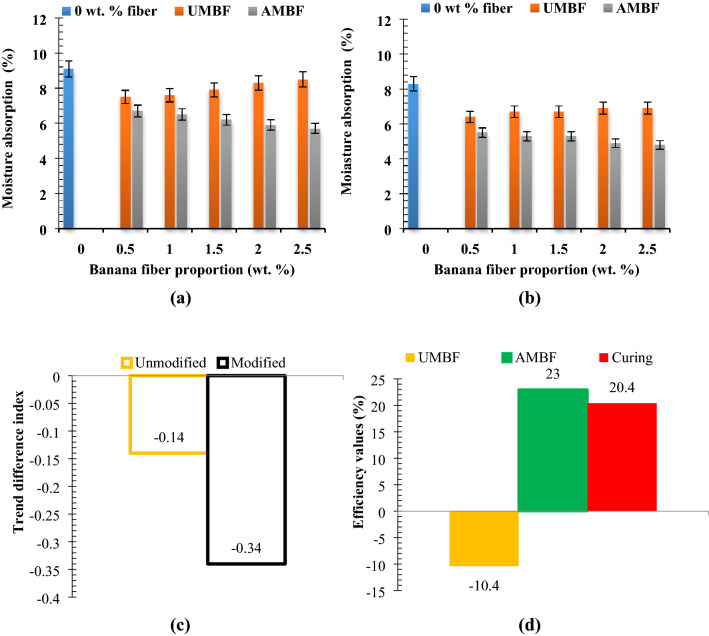
Relation between fiber proportion of unmodified and modified fiber and moisture absorption at curing days of (**a**) 28 days and (**b**) 56 days with (**c**) experimental trend analysis and (**d**) property evaluation of experimental variables.

Moisture absorption is an important property to be investigated in fiber-paperbricks owing to the hygroscopic nature and hydrophilicity of paper and fiber since moisture suction in paperbricks affect the durability and aesthetic of the bricks. Moisture (water vapor) absorption from open-air is a function of capillary suction occasioned by the volume of voids on the surface of the samples. Moisture ingestion was discerned to reduce from 9.1% for reference bricks by 17.6% when 0.5 wt% of UMBF was added, further inclusion of the fiber yielded a progressive rise in the value. UMBF been a natural fiber has an affinity for vapor absorption, hence increasing presence in paper matrix evokes a tendency for more moisture ingestion. On the other hand, fiber treatment resulted in depreciation in water vapor absorption tendency with AMBF loading up to 2.5 wt% (Fig. 5a). Under 49 days curing age, moisture absorption was noted to decrease by 22% and 34% at 0.5 wt% UMBF and AMBF content, respectively (Fig. 5b). It was further observed that moisture absorption increased marginally with further addition of UMBF, while in the case of AMBF moisture absorption decreased marginally down the trend with a gradual rise in AMBF content. Thus, curing day and fiber treatment minimize moisture absorption tendencies owing to reduced void diameter^59,60^.

- Experimental trend analysis and performance evaluation on moisture absorption

In Fig. 5c, the trend direction index was + 0.43 and + 0.43 for untreated banana fiber cured for 28 days and 56 days respectively, which affirms increasing fiber proportion ensued uptrend in moisture assimilation. Values for treated banana fiber cured for 28 days and 56 days are − 0.65 and − 0.77, respectively, insinuating a downtrend in moisture absorption with rising fiber content. The trend difference index was − 0.14 [82° acw] and − 0.34 [152° cw] for UMBF and AMBF signifying depreciation in moisture absorption when analyzing from 28 to 56 days. Figure 5d illustrates that UMBF addition efficiency is 10.4% [ −] depicting the fact that unmodified banana fiber inclusion contributed to moisture absorption (not favorable to the course of this study since moisture minimization is the aim, explaining why the efficiency is negative). At the same time, AMBF had an efficiency of 23% [ +] which implies that alkaline treatment contributed significantly to lowering moisture absorption by 12.6% [ +]. Curing length efficiency of 20.4% [ +] also depicts that curing of samples is efficient in reducing moisture absorption. From the values, it can be deduced that AMBF is more effective in decreasing moisture absorption than curing, although the two can be employed simultaneously.

### Compressive strength

**Figure 6:**
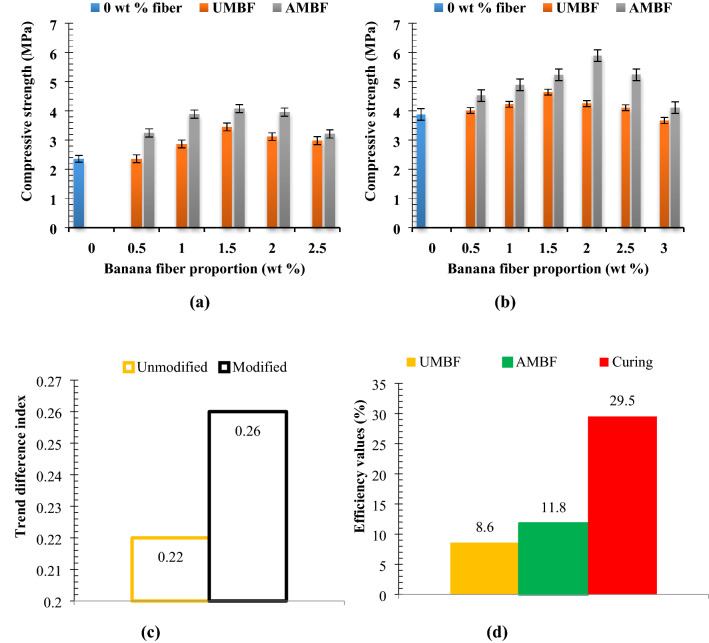
Relation between fiber proportion of unmodified and modified fiber on compressive strength at curing days of (**a**) 28 days and (**b**) 56 days with (**c**) experimental trend analysis and (**d**) property evaluation of experimental variables.

Fiber generally have been employed in previous studies to strengthen materials^61–65^, in which the increase in compressive strength was attributed to the crack arresting ability and delayed fracture mode exhibited by the fibers^66^. Fiber modification by alkaline treatment improves wettability and interfacial adhesion to the matrix, thereby enhancing the properties exhibited by the fiber. The effect of banana fiber addition on the strength of samples for both unmodified and modified banana fiber is represented in Fig. 6a,b. As the fiber content varied from 0.5 to 1.5 wt% the strength value increased progressively for samples cured under both 28 days and 56 days. The increase is a result of enhanced adhesion of fibers to the cellulose content of the paper, effectuating higher bond strength. Presence of banana fiber which served as a crack arrester boosted strength. Alkali treatment promotes interfacial bonding occasioned by increased hydroxyl density and surface charge after treatment^67^ improving mechanical strength. Further increment of 2 to 2.5 wt% of the fiber resulted in depreciation in strength which is traceable to fiber entanglement. Strong interfacial adhesion of the fiber to the matrix promotes even stress distribution within the matrix, however fiber entanglement initiates regions of stress concentration thereby imparting a measure of brittleness within the matrix, engendering depreciation in compressive strength. The reduction can also be a result of accentuation of porosity under the higher volume of fiber which could not be filled by hydrates since the cement proportion remains constant for all samples^68^. During the test, it was observed that failure was gradual and progressive. Additionally, eventual failure was more delayed in samples doped with fiber than the reference sample (0 wt% fiber).

Cement, being a strengthener contributed to the strength recorded especially at a higher curing age of 56 days, attributable to the strong hydrants formed during the hydration process. This is buttressed by the results obtained in^69–71^ indicating increased compressive strength with increased duration of curing. The highest value of compressive strength was attained at 1.5 wt% at AMBF (56 days curing) loading, an improvement of 26% relative to the reference brick. Taking a critical look at Fig. 6a,b, it is clear that there is a difference in the value of compressive strength between UMBF and AMBF with increasing fiber content. The treated fibers gave higher values when compared with their counterparts (UMBF reinforced samples). The implication of this is that alkaline modification plays a major role in the improvement of compressive strength. Results shown are in tandem with^68,71^ in which the compressive strength rose with curing age. Submission of^72,73^ affirms the findings made in this study as per rise in compressive strength with incorporation of 0–1.5 wt% fiber. It is shown in this study that natural fiber impact compressive strength. Other studies recorded similar effects, for instance, infusion of coir fiber in varying proportions of 0.5 to 4.5% at 0.5% interval revealed an upswing in compressive strength^74^ occurrence similarly reported in^75^ where coir fiber was also employed. NaOH modified coir fiber in^76^ led to enhancement of compressive strength over curing days of 7, 28, and 60 days when compared with untreated counterparts. Surface modification of kenaf fiber was recorded to yield a 42.1% improvement of compressive strength as compared with the untreated cement composites^77^. Therefore, natural fiber has the potency of enhancing the compressive strength of cement composites with modified fiber performing better. There is no standard for the compressive strength of paper-based bricks, howbeit in line with^78^ requirement for conventional bricks samples doped with 1.5 to 2 wt% AMBF met the requirement for low-cost housing. 28-day compressive strength over 6 MPa for paperbricks was attained in^79^, a value which was almost realized at 2 wt% addition of AMBF cured under 56 days.

- Experimental trend analysis and performance evaluation on compressive strength

Directional movement of property values is dependent on the influence of experimental inputs; in this case fiber addition, alkaline modification, and curing length (duration of curing). Evaluation of experimental variables as they affect the outcome of the result is as indicated in (Fig. 6c,d). Trend direction for UMBF-28 reinforced samples gave a direction index of + 0.21, implying an upward trend and a positive trend generator. Meanwhile, the trend direction index of AMBF-28 reinforced samples was + 0.42 as highlighted in Fig. 6c. The trend direction index for 56 days was calculated to be + 0.43 for UMBF and + 0.68 for AMBF. Trend direction differences of + 0.22 [69° cw] and + 0.26 [35° cw] were noted for UMBF and AMBF while considering the difference from 28 to 56 days. The relevance of the values is that the compressive strength had a positive directional index from 0 to 56 days for both UMBF and AMBF reinforced fibers.

UMBF addition efficiency (marginal) was 8.6% [ +] indicating fiber treatment was 8.6% effective in improving compressive strength. In the same vein, AMBF was 11.8% [ +] effective in improving compressive strength. Comparing the two values for fiber addition and alkaline modification of fiber, it is evident that the treatment of fiber has more impact in improving the compressive strength due to enhanced wetting, leading to stronger interfacial bonding compared to untreated counterpart, that is AMBF was 3.2% [ +] efficient over UMBF, implying alkaline efficiency of 3.2 [ +]. Curing duration (length) was evaluated to be 29.5% [ +] efficient in improving compressive strength. The significance of this is that the curing length is the most effective of the three experimental variables when improving compressive strength. The efficiencies are noted positive [ +] based on the fact that increased compressive strength is favorable in this study for paperbricks.

### Flexural strength

**Figure 7:**
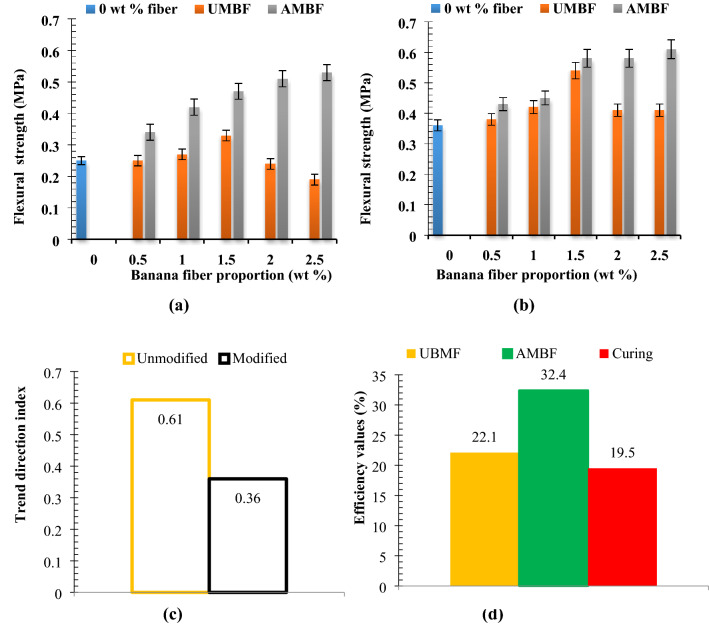
Relation between fiber proportion of unmodified and modified fiber on flexural strength at curing days of (**a**) 28 days and (**b**) 56 days with (**c**) experimental trend analysis and (**d**) property evaluation of experimental variables.

The responses of the composites to loads applied in determining the flexural strengths are as shown in Fig. 7 for UMBF and AMBF reinforced composites. Flexural strength was discerned to improve for both UMBF and AMBF inclusion in the range of 0 wt% to 1.5 wt% for samples cured for 28 and 56 days. Immix of 2 and 2.5 wt% UMBF led to depreciation in strength by 27.2 and 42.4% (Fig. 7a) respectively, relative to the value obtained at 1.5 wt% UMBF content.). 56-day flexural strength was also noted to peak on inclusion of 1.5 wt% UMBF while further inclusion of 2 wt% UMBF led to a decrease in strength relative to peak value obtained when 1.5 wt% UMBF was incorporated (depreciation of 24%). The value was maintained on infusion of 2.5 wt% UMBF (Fig. 7b). Reason for the reduction in strength value at 2 and 2.5 wt% UMBF in composite mix can be linked to reduced interfacial bonding at that wt% owing to the large volume proportion of fiber and possible fiber disorientation during loading. Flexural strength showcased in^80–83^ showed that sisal, basalt, pineapple leaf, and coir fibers, improve flexural strength in cement composites similar to the performance of banana fiber accounted for in the present investigation.

AMBF-28 days and AMBF-56 days presented a different result in the sense that the strength appreciated progressively as fiber content increased and peaked at 0.53 and 0.61 MPa, respectively. The progressive increase in the strength from 0 wt% to 2.5 wt% for AMBF-28 days and AMBF-56 days occurred on the dint of fiber freeness owing to fiber surface modification, suggesting that the fiber treatment improves flexural strength. Flexural strength reported in this study follows almost the same trend reported in^84^ and contrary to^85,86^ for additives incorporated and noted to increase with curing days as expressed in^87,88^.

- Experimental trend analysis and performance evaluation on flexural strength

Figure 7c demonstrated the trend direction indices for flexural strength at 28 and 56 days. Trend direction index for unmodified fibers reinforced samples cured for 28 and 56 days are 0 and + 0.41, while for modified, the values are + 0.61 and + 0.77 respectively. The values affirmed that blending of UMBF and AMBF in the range of 0 to 2.5 wt% illustrated an upward trend in flexural strength for reference bricks. Trend difference index was + 0.61 [90° cw] and + 0.36 [49° cw], respectively, for UMBF and AMBF emphasizing that over 56 curing days, flexural strength was improved and trended upward.

Performance of fiber, alkaline modification, and curing length was evaluated. 22.1% [ +], 32.4% [ +], and 19.5% [ +] were recorded for UMBF efficiency, AMBF efficiency, and curing length efficiency, respectively, as indicated in Fig. 7d. Alkaline modification, therefore, was assessed to be 10.3% [ +] that is AMBF was 10.3% efficient over UMBF in improving flexural strength. Deduction made from this is that fiber addition, alkaline modification, and curing length resulted in improvement in flexural strength which is favorable for this study, hence, all denoted positive. Alkaline modification mostly contributed to the feat achieved for flexural strength properties based on enhanced interfacial adhesion and improved bond strength as reported by the efficiency of AMBF.

### Splitting tensile

**Figure 8:**
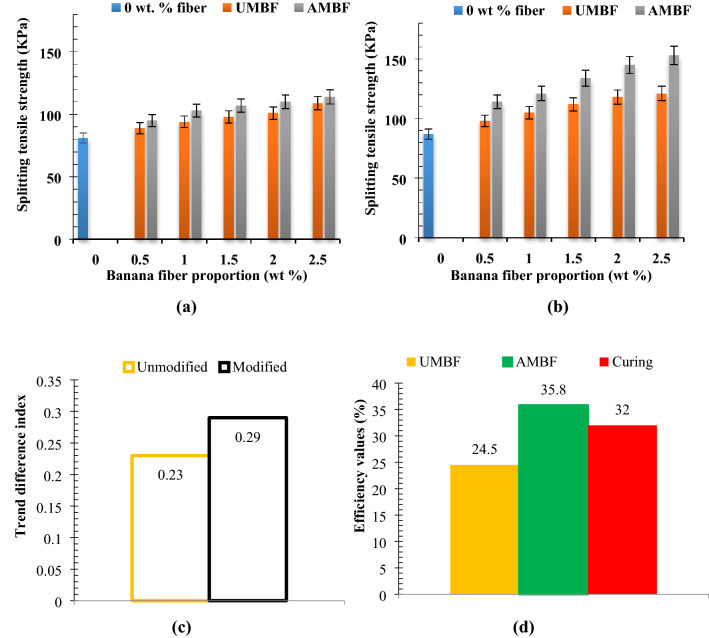
Relation between fiber proportion of unmodified and modified fiber on splitting tensile strength at curing days of (**a**) 28 days and (**b**) 56 days with (**c**) experimental trend analysis and (**d**) property evaluation of experimental variables.

Effects of fiber addition for both treated and untreated banana fibers on splitting tensile carried out on the composite are as shown in Fig. 8a,b while the experimental trend analysis is as shown in Fig. 8c. It was noted that the splitting tensile strength increased progressively as the weight % fiber inclusion increased from 0 wt to 2.5 wt% for both treated and untreated fibers (Fig. 8a). The improvement is reflected in the two curing ages (28 and 56 days) as expressed in Fig. 8a,b, and this is in connection with enhanced bond strength and interfacial adhesion. Fiber presence impacted some plastic deformation behavior within the matrix before complete fracture. As a result of the occurrence of plastic deformation (even in a small amount compared to ductile materials), toughness is enhanced in ceramics thereby extending the time to fracture^89^. Similar work reported in^90–92^ observed an increased value in splitting tensile with a proportional increase in additives. Moreover, outcomes highlighted in^93–95^ show that splitting tensile strength is appreciated with longer curing age, a fact confirmed in this study. Treated bamboo fiber employed by researchers in^96^ revealed that alkali treatment of the fiber-enhanced split tensile strength over the untreated counterpart, similar to the report pinpointed in the present investigation.

- Experimental trend analysis and performance evaluation on splitting tensile

Splitting tensile increased with fiber incorporation under 28 days curing for samples containing the two forms of banana fiber (UMBF and AMBF) involved in this study. Trend direction indices for UMBF-28 and AMBF-28 are + 0.25 and + 0.59 which denotes AMBF yielded higher strength value than UMBF. Splitting tensile enhancement was more pronounced at 56 days curing than 28 days curing as depicted in trend direction index of + 0.48 and + 0.88 for UMBF-56 and AMBF-56 accordingly. Trend difference of + 0.23 [245° cw] and + 0.29 [188° cw] was recorded for UMBF and AMBF indicating a positive trend generator further affirming the influence of fiber addition in enhancing splitting tensile strength. The performance of the experimental variables (fiber addition, alkaline modification, and curing length/duration) which determined the outcome of the results (Fig. 8d) was further evaluated. Efficiencies for UMBF addition, AMBF addition, and curing duration was 24.5% [ +], 35.8% [ +], and 32% [ +], respectively. From this, AMBF was more effective in enhancing splitting tensile strength by 11.3% [ +] denoted as alkaline efficiency. The positive signs signify a higher value for splitting strength as contributed by the experimental variables which is favorable for this study. AMBF produced the highest contribution based on the strong bond within the matrix, hence alkaline modification is highly significant for the enhancement of flexural strength.

### Thermal conductivity

**Figure 9:**
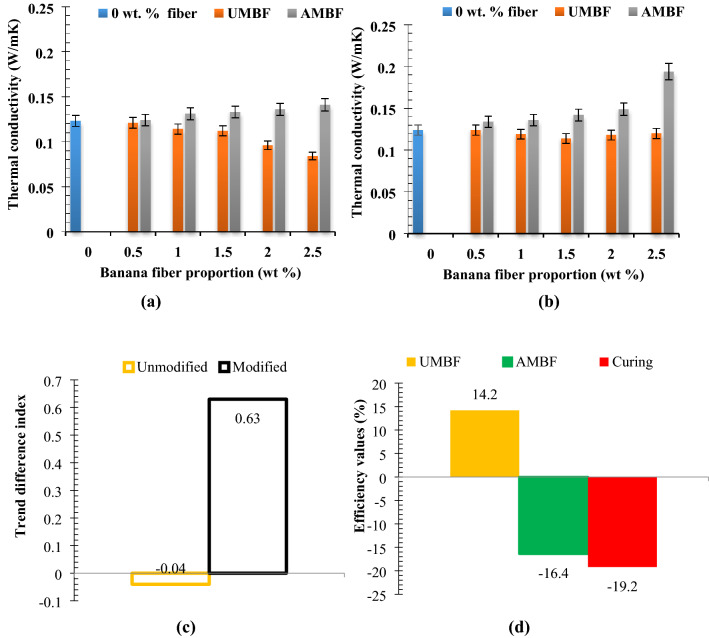
Relation between fiber proportion of unmodified and modified fiber on thermal conductivity at curing days of (**a**) 28 days and (**b**) 56 days with (**c**) experimental trend analysis and (**d**) property evaluation of experimental variables.

Thermal conductivities obtained for the samples evinced UMBF addition resulted in a downward trend relative to reference brick as shown in Figs. 9a–d respectively. Appreciation in the proportion of natural fiber content contributes to poor conduction properties of materials owing to the lower conductivity of natural fibers^97^. Opposite behaviour was exhibited by AMBF as thermal conductivity trended upward with modified fiber loading. This is attributable to the higher surface charge, crystallinity, and surface reactivity of the fibers^98,99^. The values for untreated remained almost constant with no significant change under 56 days curing age similar to what was obtained in^100^, while the values of treated improved. Another natural fiber (millet fiber) featured in^101^ demonstrated a similar result exposed in this study. From the results obtained it can be inferred that curing age has a significant effect on thermal conductivity. As per^102^, all samples satisfied the requirement since the thermal conductivity is lesser than 0.6 W/mK.

- Experimental trend analysis and performance evaluation on thermal conductivity

Experimental trend analysis is as shown in Fig. 9c,d from where it can be seen that thermal conductivity initially reduced from 0 to 2.5 wt%. UMBF-28 gave a directional index of − 0.35 (downtrend) and that of UMBF-56 was − 0.32 (downtrend). AMBF-28 had a directional trend index of + 0.31 (uptrend) and for AMBF-56 it was + 0.34 (uptrend). The resultant trend difference for UMBF is − 0.04 [75° acw, 2 cycles] which implies the effect of hydration over 56 days overshadowed the influence of UMBF in reducing thermal conductivity. The resultant trend difference for AMBF over 56 days is + 0.63 [280° acw] which delineates that thermal conductivity was enhanced over the curing days. Analysis of performance shows that the UMBF efficiency is 14.2% [ +] (Fig. 9d); that is, the untreated fiber contributes to the reduction in thermal conductivity which is favorable for thermal insulating bricks, hence a positive [ +] sign is attached. AMBF efficiency is 16.4% [ −], insinuating that alkaline modification resulted in a rise in thermal conductivity which is not favorable for the application. Alkaline efficiency is 2.2 [ −] while curing efficiency is 19.2% [ −]; connoting that the alkaline and curing length increased the thermal conductivity and not favorable for this application (reason for the negative sign).

### Specific heat capacity

**Figure 10:**
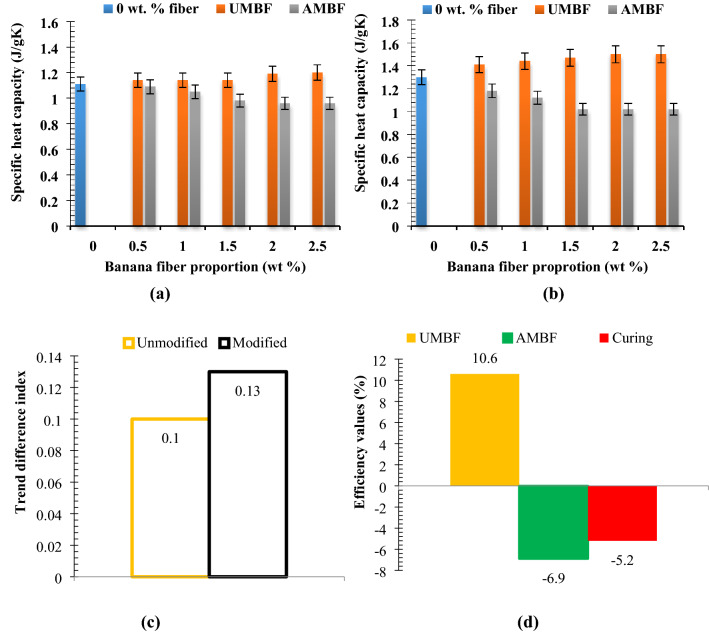
Relation between fiber proportion of unmodified and modified fiber on specific heat capacity at curing days of (**a**) 28 days and (**b**) 56 days with (**c**) experimental trend analysis and (**d**) property evaluation of experimental variables.

A higher specific heat capacity would be good for paperbricks. From Fig. 10a,b, it was noted that the specific heat capacity at 0.5 wt% UMBF increased by 2.7% when compared with reference brick and remained constant at that value as 1 and 1.5 wt% UMBF were infused. Blending of 2 and 3 wt% UMBF yielded an 8.1 and 9% rise in property value. In general, the influence of UMBF on 28-day specific heat capacity is minimal. Effect of AMBF on 28-day specific heat capacity was detected to decrease progressively on account of blending of 0.5 wt% to 2 wt% AMBF, while 2.5 wt% AMBF addition ensued same property value as was obtained at 2 wt% AMBF. The reduction was 1.8, 5.4, 11.7, 13.5, and 13.5% which shows that AMBF had a significant contribution in lowering heat capacity. This is based on the fiber freeness and increased charge which had a lowering effect on the heat capacity. Analysis of 56-day specific heat capacity (Fig. 10b) shows that UMBF addition to paperbrick composite aroused consistent and progressive increment in heat capacity up to 2 wt% UMBF, of which the value was maintained when 2.5 wt% UMBF was included. With rising AMBF, the value reduced from 0.5 wt% down to 1.5 wt%, where it remained constant despite further addition of AMBF.

- Experimental trend analysis and performance evaluation on specific heat capacity

From Fig. 10c,d, the potency of fiber addition, alkaline modification, and curing duration of samples can be evaluated. Trend direction index for UMBF-28 gave a directional index of + 0.13 while AMBF-28 had a value of − 0.43. The values depicted an uptrend for UMBF and a downtrend for AMBF under 28 days curing age. Trend directional index for UMBF and AMBF under 56 days curing age was + 0.23 and − 0.56, respectively, and this also depicted an uptrend and downtrend, respectively. Trend difference index was + 0.1 [55° cw, 2 cycles] for UMBF and + 0.13 [48° cw] for AMBF. UMBF efficiency was 10.6% [ +], AMBF was 6.9% [ −] efficient, and curing length efficiency was 5.2% [ −]. When comparing the performance of the two kinds of fiber on heat capacity, it can be stated that UMBF performed positively in increasing specific heat capacity which is favorable for this study while AMBF was negative. Further analysis reflected alkaline efficiency of 3.7 [ −] picturing a point; for maximization of specific heat capacity of paperbricks, alkaline treatment is not favorable. Therefore, UMBF addition is effective in achieving a considerable increase in specific heat capacity as needed in the paper-cement composite for masonry.

### Microstructural analysis

**Figure 11:**
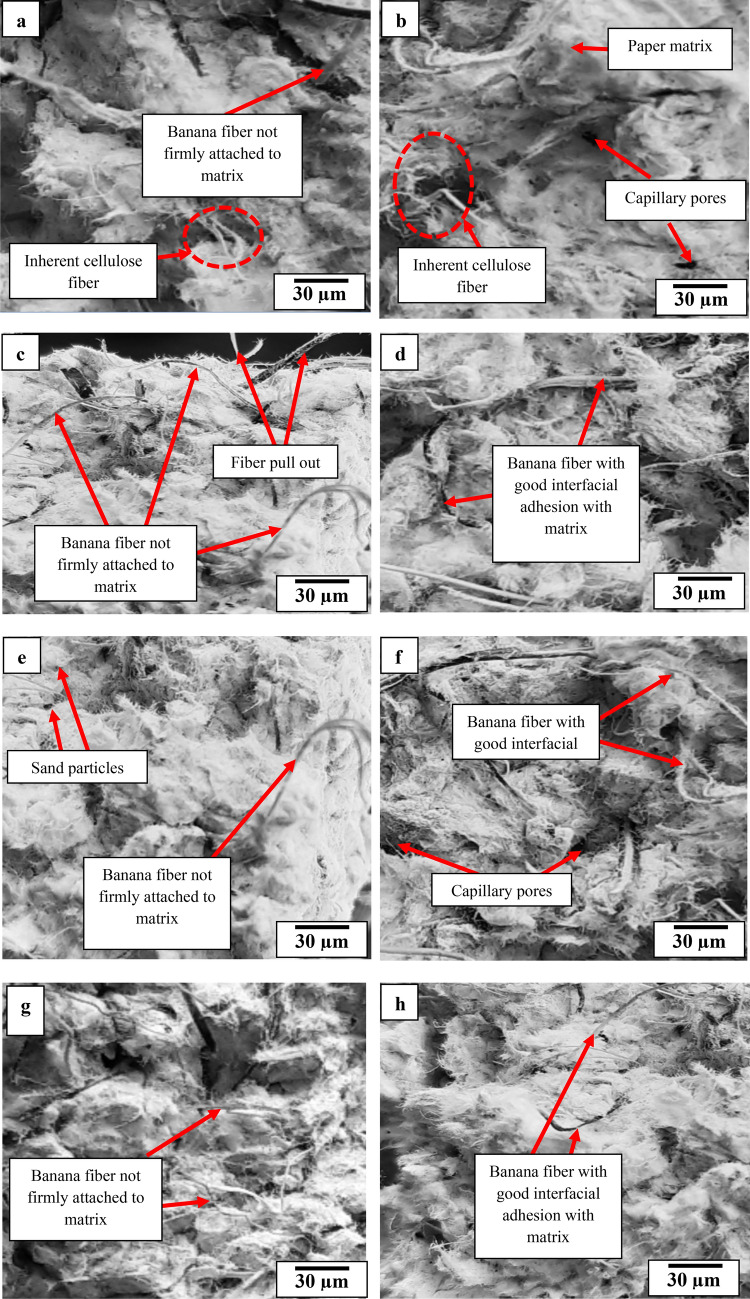

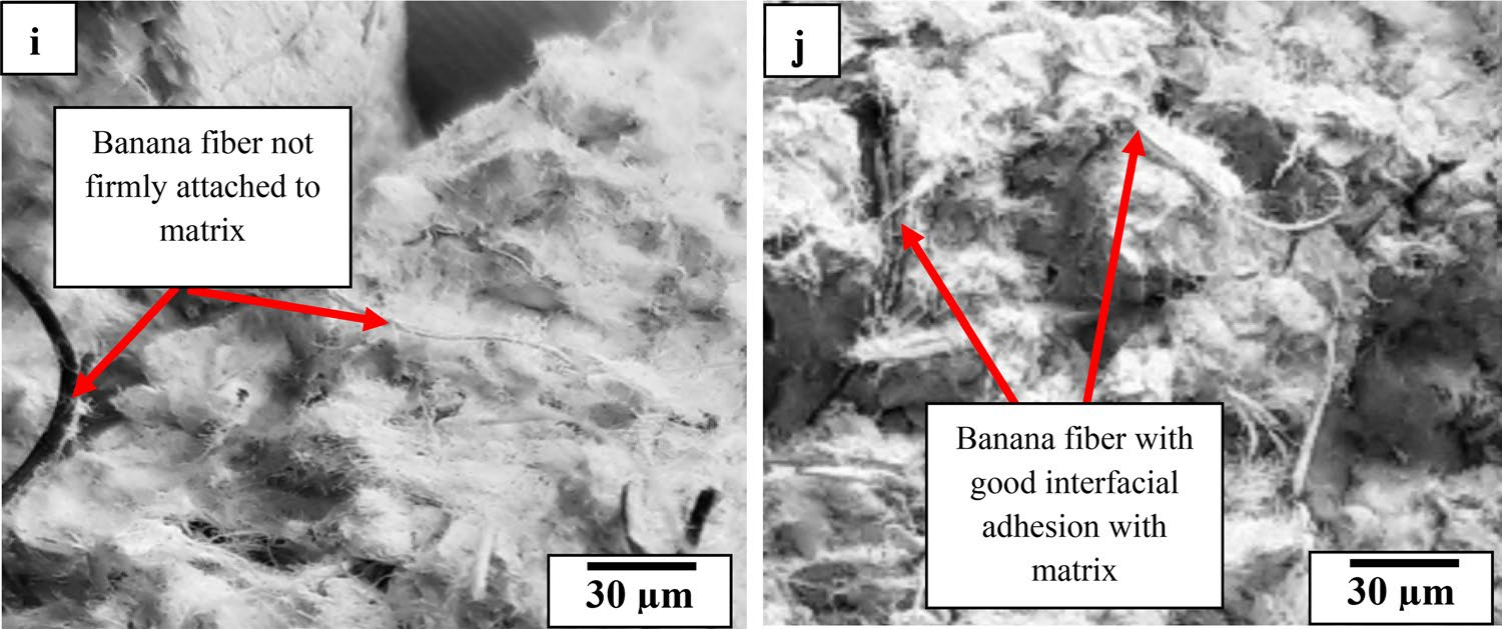
Scanning electron micrograph images of paperbricks (cured for 56 days) containing varying amount of unmodified banana fiber (0.5, 1.0, 1.5, 2.0, and 2.5 wt%) represented in (**a**,**c**,**e**,**f**,**g**,**i**) respectively and modified banana fiber (0.5, 1.0, 1.5, 2.0, and 2.5 wt%) represented in (**b**,**d**,**f**,**h**,**j**).

Figure 11a,c,e,f,g,i represent SEM images of UMBF-reinforced paperbricks, in which it was observed that fibers not firmly attached to the matrix were identified. On the other hand, Fig. 11b,d,f,h,j revealed the images of paperbrick samples reinforced with AMBF reflecting strong adhesion with the matrix. This distinction was pronounced and resulted in lesser water absorption and lower reduction in moisture absorption tendency for AMBF-paperbricks. Similarly, the AMBF-paperbricks were superior to UMBF-paperbricks for compressive strength, splitting tensile, and flexural strength based on the stronger interfacial adhesion to the matrix which promoted internal bond strength. This superior performance is also reflected in thermal conductivity trending downwards and specific heat capacity trending upward in AMBF–paperbricks; properties beneficial for masonry application unlike the UBMF where thermal conductivity increased and specific heat capacity reduced.

## Conclusions

Fiber paper brick was developed by the incorporation of modified and alkaline (1 M NaOH) modified banana fiber and fine sand into cement-paper-sand mix and the samples produced were examined and reported. The following conclusions were observed.

1. water absorption increased slightly with banana fiber loading, howbeit samples infused with modified fibers absorbed lesser water volume than the unmodified ones. Moisture absorption increased with rising UMBF dosage while consecutive addition of AMBF resulted in a decrease in moisture assimilation
2. mechanical strengths were boasted with fiber inclusion even as the modified fibers presented higher enhancement. Curing duration also participated effectively in strength enhancement.
3. alkaline modification and curing were noted to be effective in improving the properties and performance of paperbricks, and therefore recommended for property optimization in paperbricks.
